# Optical Coherence Tomography Imaging of Choroidal Abnormalities in Neurofibromatosis Type 1

**DOI:** 10.1155/2013/292981

**Published:** 2013-04-22

**Authors:** Shinji Makino, Hironobu Tampo

**Affiliations:** Department of Ophthalmology, Jichi Medical University, 3311-1 Yakushiji, Shimotsuke, Tochigi 329-0498, Japan

## Abstract

We report a case of neurofibromatosis type 1 (NF1) examined by infrared fundus autofluorescence (IR-FAF) and optical coherence tomography (OCT) to characterize the associated choroidal abnormalities. The conventional ophthalmoscopic findings were unremarkable. However, IR-FAF revealed multiple bright patchy lesions in the choroid of the posterior pole, in both eyes. OCT demonstrated irregular hyperreflectivity at the sites of these lesions. Patients with NF1 may have typical choroidal lesions that are visible on IR-FAF, which can be confirmed through OCT.

## 1. Introduction

Neurofibromatosis type 1 (NF1), an autosomal dominant disorder with a high mutation rate, is considered a neurocristopathy characterized by pathological hamartomatous proliferations of neural crest-derived tissues. A minimum of 2 of the following criteria are required for diagnosis: 6 or more café-au-lait spots, 2 or more cutaneous neurofibromas, 1 or more plexiform neurofibromas, axillary or groinal freckling, optic glioma, 2 or more iris Lisch nodules, distinctive bony lesions, and a first-degree relative with NF1 [1]. Among these criteria, iris Lisch nodules are frequently observed and well recognized. However, retinal and choroidal lesions have been considered unusual in eyes with this disease.

In 2000, Yasunari et al. [2] suggested that choroidal abnormalities were easily detectable by infrared light examination with a scanning laser ophthalmoscope in 100% of their NF1 patients. Recently, the cutoff value for choroidal nodules detected by infrared fundus autofluorescence (IR-FAF) was reported to be 1.5 [3]. The choroid is one of the most commonly affected structures by NF1, and IR-FAF is typically used to detect choroidal nodules in NF1 patients [4–8]. To our knowledge, there are few reports in the literature describing the use of optical coherence tomography (OCT) to identify choroidal abnormalities in NF1 patients [3, 7, 8]. Herein, we report on the IR-FAF and OCT findings for a patient with NF1.

## 2. Case Report

**Figure 1:**
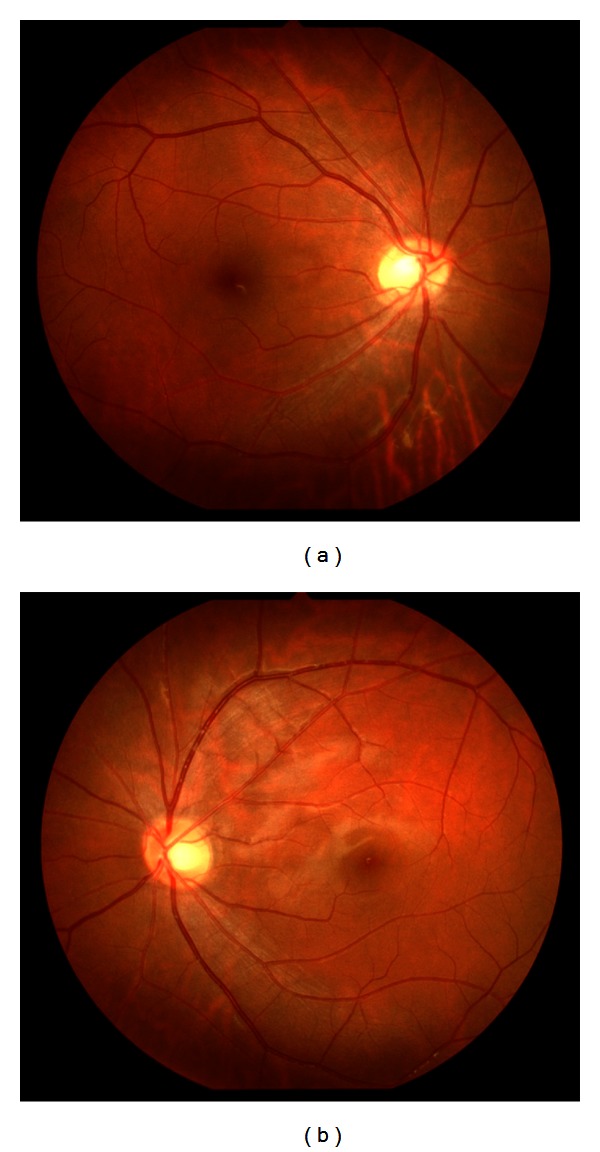
Right (a) and left (b) fundus photographs showing no abnormalities.

**Figure 2:**
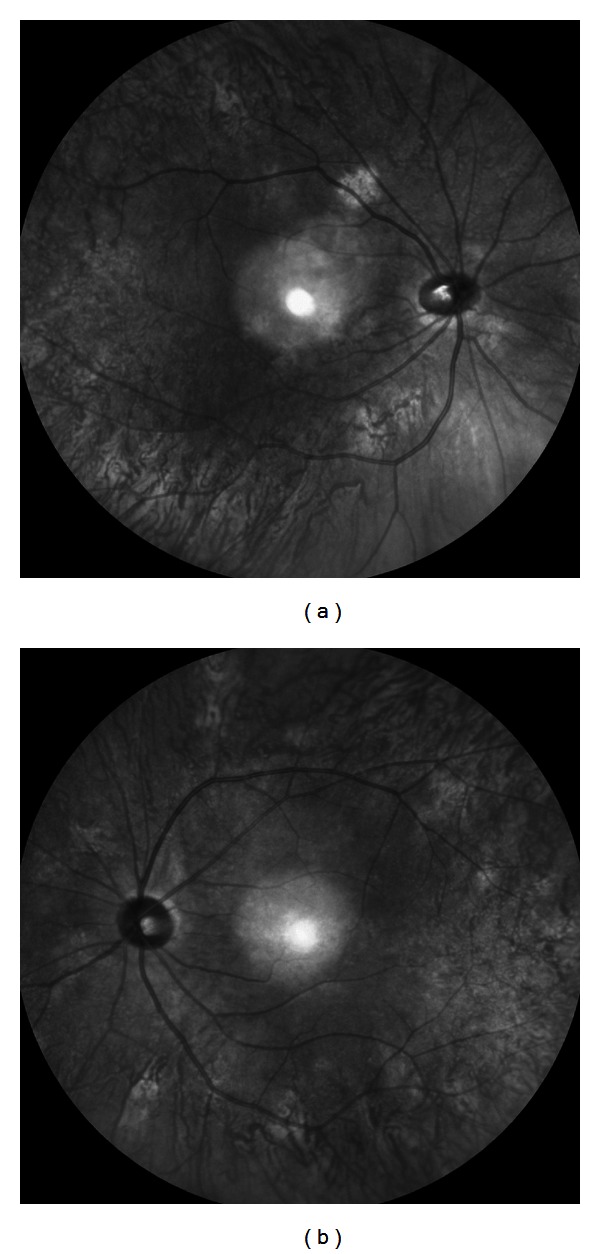
Right (a) and left (b) infrared fundus autofluorescence images. Note multiple bright, patchy lesions in the posterior poles of both eyes. The hyperreflective point at the center of the image is an optical artifact.

**Figure 3:**
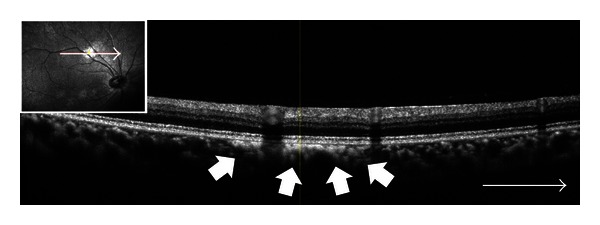
Right optical coherence tomography image. Note the irregular hyperreflectance focus in the choroid (arrows).

A 25-year-old man with NF1 was referred to our clinic for an ophthalmological examination. The NF1 diagnosis was made on the basis of several café-au-lait spots and cutaneous neurofibromas. The patient's family and personal medical history added no significant information. He had no visual symptoms. His best-corrected visual acuity was 1.2 in both eyes. There were at least 3 Lisch nodules on each side. Ophthalmoscopic examinations of the fundi did not show any abnormalities (Figures 1(a) and 1(b)). However, IR-FAF (Heidelberg Retina Angiograph 2, Heidelberg Engineering, Heidelberg, Germany) revealed multiple bright, patchy lesions in the posterior pole of the choroid. These lesions were much more prominent in the right eye than the left (Figures 2(a) and 2(b)). OCT (RS-3000, NIDEK, Japan) images of these lesions revealed irregular, hyperreflective choroidal foci (Figure 3). OCT revealed no abnormality of the outer retina.

## 3. Discussion

The ophthalmic examination revealed findings similar to those reported previously for NF1 patients [2–8]. Although the conventional ophthalmoscopic examination was normal, IR-FAF revealed multiple bright, patchy lesions. Yasunari et al. [2] suggested that choroidal abnormalities (100%) occurred more frequently than Lisch nodules in the iris (76%). They also suggested that bright patchy choroidal lesions should be a new diagnostic criterion for NF1. In a recent study with a large number of NF1 patients, Viola et al. [3] reported that IR-FAF imaging revealed choroidal nodules in 82% of NF1 patients as compared with 7% of healthy patients. The highest accuracy was obtained using a cutoff value of 1.5. 

Infrared light penetrates deeper into the eye than does visible light. It has been reported previously that IR-FAF can be used to visualize the retinal pigment epithelium (RPE) and to a varying degree, melanocytes in the choroid [9]. Similarly, melanin in human skin is reported to increase the backscattering of infrared light, such that the cytoplasm in heavily pigmented cells images brightly under confocal scanning laser microscopy [10].

In NF1 patients, the choroid hosts a proliferation of neural crest-derived melanocytes and neural cells, which results in apparent thickening of the posterior fundus [5]. A. Kurosawa and H. Kurosawa [11] reported that choroidal neurofibromatosis, as observed in an enucleated eye, was characterized by ovoid bodies consisting of proliferating neoplasmic Schwann cells that were arranged in concentric rings around the axons.

The increased choroidal thickness at the posterior pole also means an increase in the number of melanocytes and neural cells [5]. Of the 2 proliferating cell types (i.e., melanocytes and neural cells), melanocytes are rich in melanin, which absorbs near-infrared light. This results in dramatic backscattering, as predicted by the Mie scattering theory [5]. Choroidal lesions comprise densely packed, proliferated melanocytes and neural cells, which means a reduced volumetric percentage of blood components including hemoglobin, oxygenated hemoglobin, and water. Because these 3 blood components can absorb scattered near-infrared light, their relative paucity might cause the choroidal lesions to appear brighter than the surrounding choroid [5].

To our knowledge, there are few reports in literature that characterize choroidal abnormalities as detected by OCT [3, 7, 8]. In our case, OCT revealed irregular hyperreflectivity that colocalized with the choroidal lesions. Ueda-Consolvo et al. [8] described choroidal abnormalities as examined by OCT in 3 cases. Numerous previous reports have described this phenomenon [3, 7]. However, hyporeflective foci have also been observed. The authors speculated that long-standing choroidal nodules might induce focal choriocapillary atrophy, resulting in hyporeflectivity of the choroid on OCT images. Nonetheless, the outer limiting membrane and inner segment/outer segment line appeared normal when imaged using OCT. These results suggest that choroidal abnormalities in NF1 patients localized to the choroid and did not affect the retina. 

In conclusion, patients with NF1 may have typical choroidal lesions visible on IR-FAF. OCT might be useful for confirming their location. The extent of choroidal involvement is likely to vary among patients and increase with age [5]. Long-term follow-up will be necessary to further characterize the phenomenon.
